# Chemical cross‐linking facilitates antigen uptake and presentation and provides improved protection from Mpox with a dual‐antigen subunit vaccine

**DOI:** 10.1002/mco2.70045

**Published:** 2025-01-08

**Authors:** Long Chen, Chao Shang, Zihao Wang, Mengzhu Zheng, Cuiling Zhang, Dapeng Li, Zhanqun Yang, Yuchao Dong, Yuru Xu, Yunsheng Yuan, Shiyong Fan, Wu Zhong, Jian Lin, Xiao Li

**Affiliations:** ^1^ Department of Pharmacy Peking University Third Hospital Cancer Center Peking University Third Hospital Beijing China; ^2^ Changchun Veterinary Research Institute Chinese Academy of Agricultural Sciences Changchun China; ^3^ National Engineering Research Center for the Emergency Drug Beijing Institute of Pharmacology and Toxicology Beijing China; ^4^ Key Laboratory of Tropical Biological Resources of Ministry of Education Song Li's Academician Workstation of Hainan University School of Pharmaceutical Sciences Hainan University Haikou China; ^5^ Engineering Research Center of Cell & Therapeutic Antibody Ministry of Education Shanghai Jiao Tong University School of Pharmacy Shanghai China

**Keywords:** antigen uptake and presentation, chemical cross‐linking, dual‐antigen, monkeypox virus, subunit vaccine

## Abstract

Antigen uptake, processing, and presentation are crucial for the immune responses of protein‐based vaccines. Herein, we introduced a reversible chemical cross‐linking strategy to engineer protein antigens, which can be tracelessly removed upon antigen‐presenting cell (APC) uptake and cellular reduction. The chemically cross‐linked antigen proteins presented significantly enhanced uptake and epitope presentation by APC. We applied this strategy to monkeypox virus antigens A29L and A35R to construct dual‐antigen subunit vaccines. Our results revealed that chemical cross‐linking was robust enough to improve both proteins' APC uptake and lymph node accumulation, with each protein being chemically cross‐linked and administered separately. In vivo validation revealed that the chemical cross‐linking of the two antigen proteins improved immune responses, with increases in antigen‐specific antibody and live virus‐neutralizing antibody production. Monkeypox virus challenge experiments revealed that dual‐antigen vaccines prepared via the chemical cross‐linking strategy mitigated tissue damage, reduced the virus load, and extended mouse survival, which proved that the chemical cross‐linking strategy is valuable for protein‐based subunit vaccine development. In consideration of the current threats from the monkeypox virus and potential future emerging pathogens, the chemical cross‐linking strategy provide powerful tools.

## Graphical abstract

This work presented a reversable chemical cross‐linking strategy to improve the accumulation of antigens in lymph node and to increase the uptake and presentation of antigens in antigen‐presenting cell (APC). A dual‐antigen vaccine was constructed for Mpox using this strategy, which provide enhanced protection against monkeypox virus. This work thus provided universal tools for the fight against future emerging pathogens.

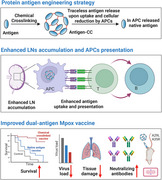

## 1 INTRODUCTION

Monkeypox (Mpox) is an animal‐origin viral disease caused by the monkeypox virus (MPXV), which belongs to the genus Orthopox in the family Poxviridae and has a genome size of approximately 197 kb.^1^, ^2^ MPXV was first discovered in 1970 and has predominantly been concentrated in Central and West Africa for the past half century, showing regional endemicity with limited human‐to‐human spread.^3^ However, since May 2022, MPXV has developed into a global pandemic, spreading to 117 countries with over 90,000 confirmed cases and 187 deaths.^4^ Thus, it poses a significant threat to global public health. Vaccines have been regarded as effective countermeasures against orthopoxvirus. Based on the similarity among the members of orthopoxvirus, the vaccinia virus (VACV) live vaccine plays an essential role in smallpox eradication.^5^, ^6^ A previous study reported that individuals subjected to a VACV live vaccine exhibited 85% protection against MPXV infection.^6^ Currently, two live attenuated VACV vaccines have been conditionally approved by the U.S. Food and Drug Administration (FDA) for preventing MPXV infection.^7^, ^8^ However, owing to the application of live pathogens, safety concerns are nonnegligible, especially considering the relatively high coinfection rate of MPXV patients with HIV and immune deficiency disorders.^9^ Thus, a safer and more effective vaccine is urgently needed to combat Mpox.

Protein‐based subunit vaccines utilize a specific antigen protein from a pathogen, such as the spike protein from SARS‐CoV‐2, to stimulate immune responses and thus offer protection from the pathogen to the host.^10^, ^11^ Unlike attenuated or inactivated vaccines, protein‐based subunit vaccines do not contain the whole pathogen, which makes them safer to use and quite easy to design with multiple formats. Despite these advantages, protein‐based subunit vaccines have their disadvantages, one of which is the low immunogenicity of the antigen proteins.^11^ Therefore, engineering antigen proteins and designing multiantigen formats are crucial for developing protein‐based subunit vaccines. In our previous work, we successfully engineered the receptor‐binding domain of the spike protein for SARS‐CoV‐2 and developed a potential subunit vaccine for COVID‐19 with elevated immune responses by cross‐linking the recombinant proteins into nanoscale material in a single‐antigen format but with incomprehensiveness in vitro and in vivo analyses and presentations.^12^ Therefore, in this work, we aimed to expand the chemical cross‐linking strategy to multiantigen subunit vaccines for Mpox with more comprehensive in vitro and in vivo analyses.

A29L and A35R are two major antigen proteins in MPXV. A29L is a membrane fusion protein that plays a critical role in the binding of MPXV to heparin on host cell surfaces, which facilitates the entry of the virus into host cells.^13^ A35R is another envelope glycoprotein vital for the formation of effective extracellular enveloped viruses (EEVs) that are responsive to the intercellular spread of MPXV.^13^ These characteristics make A29L and A35R appealing targets for developing MPXV vaccines.^14^, ^15^, ^16^, ^17^, ^18^, ^19^ However, in our initial trial in which A29L and A35R were used as recombinant antigens, we noted the limited uptake of these two proteins by antigen‐presenting cells (APCs), especially A29L, suggesting that the two antigen proteins need to be engineered to improve their uptake and presentation by APCs for better immune responses.

**FIGURE 1:**
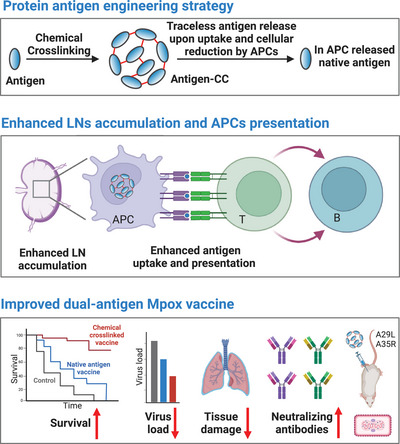
Schematic representation of the chemical cross‐linking strategy, its functions in the accumulation of antigens in lymph nodes and presentation by antigen‐presenting cells, and the development of an improved dual‐antigen monkeypox vaccine with this strategy.

In this study, we introduced a chemical editing strategy, termed chemical cross‐linking, to engineer protein antigens (Figure 1). The chemical cross‐linking strategy reversibly cross‐linked the protein antigens into larger molecular protein polymers and the uptake of the protein antigens by APCs. The protein antigen polymers subsequently break down into native antigen proteins upon cellular reduction, which avoids the blockage of the native epitopes. With the model ovalbumin (OVA) antigen, we comprehensively demonstrated that the enhanced antigen presentation resulted from the chemical cross‐linking strategy. This strategy was then applied to recombinant A29L and A35R antigen proteins to construct a dual‐antigen vaccine for Mpox. We comprehensively validated the cross‐linking formats (chemical cross‐linking separately or cooperatively) and administration methods (separately or cooperatively) and finally identified the appropriate dual‐antigen vaccine format and administration method. In this way, chemical cross‐linking improved the uptake and antigen presentation of the two proteins in APCs. In vivo validation revealed that upon immunization, the chemically cross‐linked dual‐antigen vaccine elicited high levels of specific and live virus‐neutralizing antibodies. In the virus challenge experiments, the chemically cross‐linked dual‐antigen vaccine reduced the MPXV load, mitigated tissue damage, and extended mouse survival. These results indicate that a chemical cross‐linking strategy could be applied to develop improved multiantigen protein‐based subunit vaccines.

## 2 RESULTS

### 2.1 Chemical cross‐linking facilitates antigen uptake and presentation

**FIGURE 2:**
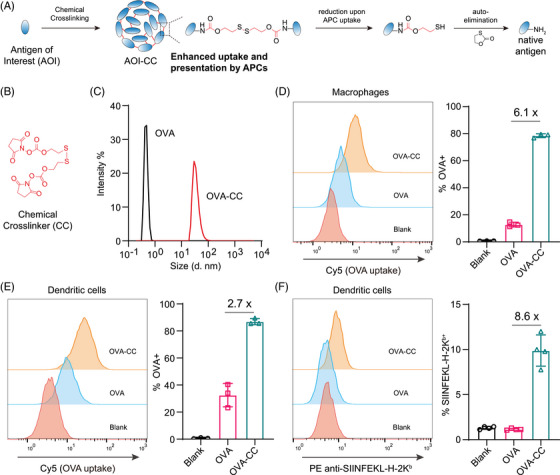
Chemical cross‐linking facilitates antigen uptake and presentation, as illustrated using a model antigen. (A) Schematic representation of the chemical cross‐linking of protein antigens and the mechanism of traceless release of native antigens. (B) Structure of the chemical cross‐linker (CC) molecule. (C) Dynamic light scattering analysis of the ovalbumin (OVA) antigen and chemically cross‐linked ovalbumin (OVA‐CC). Diameters: OVA, 0.47 nm; OVA‐CC, 35.3 nm. PDI (polydispersity index): OVA, 0.663; OVA‐CC, 0.368. (D) Flow cytometry analysis of the uptake of OVA and OVA‐CC by antigen‐presenting macrophages. Left: flow cytometry results. Right: Quantitative representation of the flow cytometry results. *n* = 3. (E) Flow cytometry analysis of the uptake of OVA and OVA‐CC by antigen‐presenting dendritic cells. Left: flow cytometry results. Right: Quantitative representation of the flow cytometry results. *n* = 3. (F) Flow cytometry analysis of antigen presentation by dendritic cells. The presentation of the ovalbumin peptide SIINFEKL was validated with an antibody targeting the SIINFEKL‐H‐2Kb complex. Left: flow cytometry results. Right: Quantitative representation of the flow cytometry results. *n* = 4. All the data are expressed as the mean  ±  SD.

We first validated the effects of chemical cross‐linking on protein antigen uptake and presentation in vitro. We adopted a chemical cross‐linking strategy that forces the formation of protein antigen nanoparticles (Figure 2A). The nanoparticles were formed by cross‐linking lysine residues with a redox‐responsive disulfide bond. Upon uptake by APCs, the disulfide bond is reduced, followed by auto‐elimination of the residue modification to release the native antigen (Figure 2A). The structure of the chemical cross‐linker molecule is shown in Figure 2B. We first used OVA as a model antigen. Dynamic light scattering (DLS) revealed that chemical cross‐linking of OVA (resulting in OVA‐CC) resulted in the formation of larger protein nanoparticles (Figure 2C). Uptake of OVA and OVA‐CC was first examined in macrophages. Compared with native OVA, OVA‐CC resulted in 6.1‐fold greater uptake, as illustrated by flow cytometry analysis (Figure 2D). The enhanced cellular uptake effect was further validated in dendritic cells. The results revealed that the uptake of OVA‐CC by dendritic cells was also 2.7‐fold greater than that of OVA‐CC (Figure 2E). We then envisioned that the increased uptake of cross‐linked antigen by APCs would result in increased antigen processing and presentation. With the model OVA antigen, the presentation of the classic SIINFEKL epitope was indeed strengthened (Figure 2F). Therefore, with the model OVA antigen, we proved that chemical cross‐linking facilitates antigen uptake and presentation; thus, the chemical cross‐linking strategy is a promising method for vaccine development.

### 2.2 Chemical cross‐linking of the MPXV antigens A29L and A35R

**FIGURE 3:**
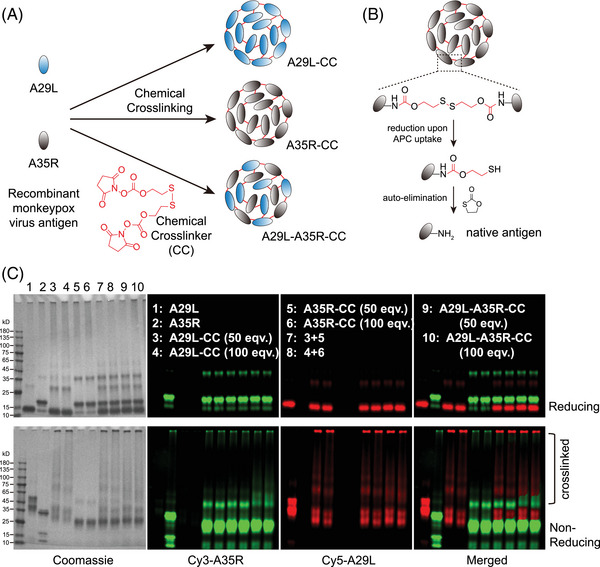
Chemical cross‐linking of monkeypox virus antigens A29L and A35R. (A) Schematic representation of the chemical cross‐linking of monkeypox virus antigens A29L and A35R separately or in combination. (B) Schematic representation of the breakdown of chemically cross‐linked antigens to release native antigens. (C) Reducing and nonreducing sodium dodecyl sulfate–polyacrylamide gel electrophoresis (SDS‒PAGE) analysis of chemically cross‐linked A29L‐CC, A35R‐CC, and A29L‐A35R‐CC (co‐cross‐linked A29L and A35R) prepared with different equivalents of chemical cross‐linker molecules. A29L and A35R were prelabeled with Cy5 and Cy3 for fluorescence visualization. Cross‐linked bands are indicated.

MPXV is another concerning virus in the post‐COVID‐19 era. A29L and A35R are two major antigen proteins utilized to develop recombinant MPXV vaccines. Inspired by the enhanced antigen uptake, processing, and presentation by chemical cross‐linking, we decided to construct a novel dual‐antigen vaccine of A29L and A35L via this chemical cross‐linking strategy. The two proteins were first expressed and purified from *E. coli* (Figure S1). The two recombinant antigen proteins were then designed to be cross‐linked individually as A29L‐CC and A35R‐CC or co‐cross‐linked as A29L‐A35R‐CC (Figure 3A). The chemically cross‐linked antigens are reduced and undergo auto‐elimination to release native antigens upon uptake by APCs (Figure 3B). Different equivalents of chemical cross‐linker molecules were used to prepare cross‐linked antigens. Reducing and nonreducing sodium dodecyl sulfate‐polyacrylamide gel electrophoresis (SDS‒PAGE) was performed to validate the cross‐linked antigens (Figure 3C). The results showed that either 50‐ or 100‐valent cross‐linker molecules resulted in efficient antigen cross‐linking with the two antigen proteins either cross‐linked alone or in combination (Figure 3C, lower panel). A29L also showed more efficient cross‐linking, consistent with the DLS results, as A29L‐CC had a larger diameter than A35R‐CC (Figure S2). Reducing with dithiothreitol (DTT) containing SDS‒PAGE loading buffer resulted in the breakdown of cross‐linked antigens and release of monomeric antigen proteins (Figure 3C, upper panel). A 50‐equivalent cross‐linker was thus selected to prepare A29L‐CC, A35R‐CC, and A29L‐A35R‐CC for subsequent experiments.

### 2.3 Chemical cross‐linking facilitates A29L and A35R uptake separately by APCs in vitro

**FIGURE 4:**
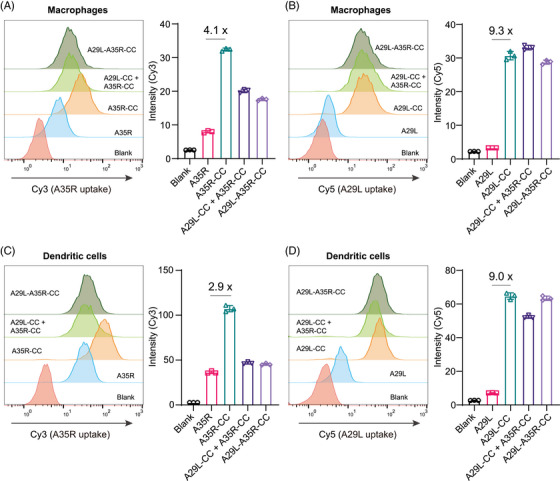
Chemical cross‐linking facilitates A29L and A35R uptake separately by antigen‐presenting cells in vitro. (A) Flow cytometry analysis of A35R uptake by dendritic cells as an A35R monomer, A35R‐CC, a mixture of A35R‐CC and A29L‐CC, or A29L‐A35R‐CC. Left: flow cytometry results. Right: Quantitative representation of the flow cytometry results. A35R‐CC alone resulted in the highest uptake by dendritic cells. *n* = 3. (B) Flow cytometry analysis of A29L uptake by dendritic cells as an A29L monomer, A29L‐CC, a mixture of A35R‐CC and A29L‐CC, or A29L‐A35R‐CC. Left: flow cytometry results. Right: Quantitative representation of the flow cytometry results. A29L‐CC alone resulted in the highest uptake by dendritic cells. *n* = 3. (C) Flow cytometry analysis of A35R uptake by macrophages as an A35R monomer, A35R‐CC, a mixture of A35R‐CC and A29L‐CC, or A29L‐A35R‐CC. Left: flow cytometry results. Right: Quantitative representation of the flow cytometry results. A35R‐CC alone resulted in the highest uptake by macrophages. *n* = 3. (D) Flow cytometry analysis of A29L uptake by macrophages as an A29L monomer, A29L‐CC, a mixture of A35R‐CC and A29L‐CC, or A29L‐A35R‐CC. Left: flow cytometry results. Right: Quantitative representation of the flow cytometry results. A29L‐CC alone resulted in the highest uptake by macrophages. *n* = 3. All the data are expressed as the mean  ±  SD.

We validated the uptake of cross‐linked A29L‐CC, A35R‐CC, and A29L‐A35R‐CC by APCs in vitro. The uptake of the antigens was validated in the forms of individual cross‐linking (i.e., A29L‐CC or A35R‐CC), dual‐antigen co‐cross‐linking (i.e., A29L‐A35R‐CC), or a mixture of individual cross‐linking (i.e., A29L‐CC+ A35R‐CC). Native A29L and A35R proteins were pre‐labeled with Cy3 and Cy5, respectively, for visualization before cross‐linking. Uptake was first validated in macrophages. Flow cytometry analysis revealed that A35R‐CC led to enhanced uptake of the A35R antigen, and quantitative analysis revealed a 4.1‐fold increase in A35R uptake compared with monomeric A35R (Figure 4A). However, beyond our expectation, either a mixture of A29L‐CC and A35R‐CC or dual‐antigen co‐cross‐linked A29L‐A35R‐CC led to a significant reduction in A35R antigen uptake compared with A35R‐CC alone (Figure 4A). This interesting finding requires further investigation to elucidate the mechanism involved. A29L uptake by macrophages were also validated. Like A35R, the uptake of A29L‐CC was significantly enhanced by 9.3‐fold compared with monomeric A29L (Figure 4B). For A29L, A29L‐A35R‐CC resulted in only a slight reduction in A29L‐CC uptake, while the mixture of A29L‐CC and A35R‐CC even led to slightly enhanced uptake by macrophages (Figure 4B), which contrasted with the results of A35R, suggesting that the chemical cross‐linking strategy may have a distinct effect on different antigens.

The uptake of these antigens was further validated in dendritic cells. Like the results in macrophages, both A35R‐CC and A29L‐CC alone increased antigen uptake (Figure 4C,D). For A35R, a mixture of A29L‐CC and A35R‐CC or A29L‐A35R‐CC led to a significant reduction in A35R antigen uptake compared with A35R‐CC alone in dendritic cells (Figure 4C), whereas only slightly A29L antigen uptake reduction was observed with the mixture of A29L‐CC and A35R‐CC (Figure 4D). The internalization of A29L‐CC and A35R‐CC by dendritic cells and macrophages was visualized via confocal microscope imaging (Figure S3‐6).

Taken together, these in vitro results suggest that in vivo immunization should be separated A35R‐CC and A29L‐CC.

### 2.4 Chemical cross‐linking facilitates A29L and A35R accumulation in inguinal lymph nodes and the induction of specific antibodies

**FIGURE 5:**
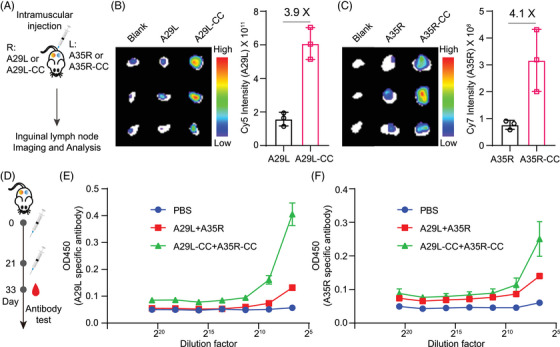
Chemical cross‐linking facilitates A29L and A35R accumulation in inguinal lymph nodes and the induction of specific antibodies. (A) Schematic representation of the characterization process of A29L/A35R or A29L‐CC/A35R‐CC accumulation in inguinal lymph nodes. (B) Ex vivo analysis of A29L accumulation in inguinal lymph nodes as an A29L monomer or A29L‐CC. Left: ex vivo fluorescence imaging. Right: quantitative analysis. *n* = 3. (C) Quantitative analysis of A35R‐ or A35R‐CC accumulation in inguinal lymph nodes. Left: ex vivo fluorescence imaging. Right: quantitative analysis. *n* = 3. The data are expressed as the mean  ±  SDs for (B) and (C). (D) Schematic representation of the immunization process involving A29L/A35R or A29L‐CC/A35R‐CC. The mice were primed and boosted with A29L/A35R or A29L‐CC/A35R‐CC separately, with each protein antigen injected intramuscularly. (E) Analysis of A29L‐specific antibodies. A chemically cross‐linked antigen resulted in a higher titer of A29L‐specific antibody. *n* = 5. (F) Analysis of A35R‐specific antibodies. A chemically cross‐linked antigen resulted in a higher titer of A35R‐specific antibody. *n* = 5. The data are expressed as the mean  ±  SEMs for (E) and (F).

We envisioned that cross‐linking would lead to enhanced accumulation of antigens in inguinal lymph nodes in vivo, as this strategy forced the formation of larger antigen formats (Figure 3). To prove this hypothesis, fluorescence‐labeled monomeric antigens or cross‐linked antigens were injected intramuscularly into mice, with each antigen administered to one of the mouse flanks separately (Figure 5A). Ex vivo fluorescence imaging revealed that compared with their monomeric antigens, both A29L‐CC and A35R‐CC led to increased accumulation of antigens in inguinal lymph nodes (Figure 5B,C). Quantitative analysis revealed 3.9‐ and 4.1‐fold increases in A29L‐CC and A35R‐CC, respectively (Figure 5B,C).

Inspired by the above results, we expected that A29L‐CC and A35R‐CC would enhance immune responses in vivo. We set up an in vivo priming and boosting immunization process to test this hypothesis by validating specific antibodies (Figure 5D). Each mouse was immunized with both antigens on one side of the hind leg area. An enzyme‐linked immunosorbent assay (ELISA) was used to test A29L‐ and A35R‐specific antibodies. The results revealed that both A29L‐CC and A35R‐CC led to increased titers of specific antibodies, especially A29L‐CC (Figure 5E,F), which agreed with the increased cross‐linking efficacy of A29L (Figure 3C). These results suggest that the chemical cross‐linking of both antigens leads to enhanced immune responses.

### 2.5 Chemically cross‐linked antigens facilitate the induction of neutralizing antibodies and reduce the virus load in vivo

**FIGURE 6:**
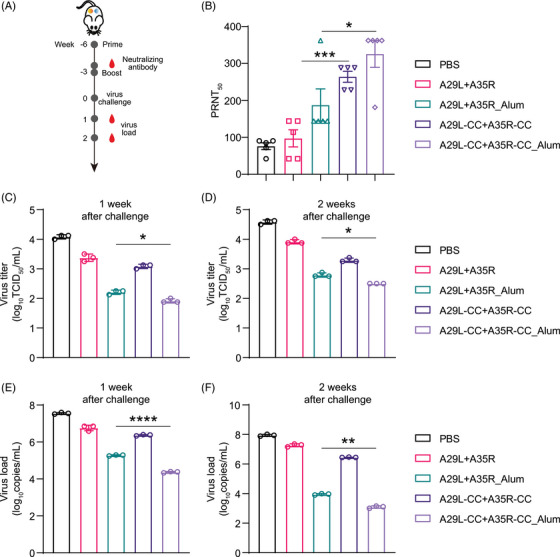
Chemically cross‐linked antigens induce neutralizing antibodies and reduce the virus load in vivo. (A) Schematic diagram of immunization, monkeypox virus (MPXV) challenge, and sample collection. Groups of dormice were immunized with vaccines or placebo and boosted with an equal dose 3 weeks later. Before the second immunization, the serum samples were collected for neutralizing antibody detection. After another 3 weeks, the immunized dormice was inoculated with 10^5.5^ MPXV via intranasal injection. The dormice were sacrificed at the indicated time points to isolate lung tissues. (B) Titers of serum‐neutralizing antibodies against MPXV were determined via the PRNT assay. *n* = 5. (C and D) Viral titers in lung tissues from MPXV‐inoculated dormice at 1 and 2 weeks post‐infection. *n* = 3. (E and F) qRT‐PCR analysis of lung tissues from MPXV‐inoculated dormice at 1 and 2 weeks post‐infection. *n* = 3. The data are expressed as the mean  ±  SEMs. *p < 0.05, **p < 0.01, ***p < 0.001.

We further determined the efficacy of our chemically cross‐linked antigens in protecting animals from MPXV infection. We designed and tested four formulations, including A29L+A35R antigens, A29L+A35R antigens combined with aluminum adjuvants (A29L+A35R_Alum), and chemically cross‐linked formulations (A29L‐CC+A35R‐CC, A29L‐CC+A35R‐CC_Alum). Initially, groups of dormice were immunized with a single dose of vaccine via i.m. inoculation, and phosphate‐buffered saline (PBS) was used as a placebo. The dormice were then boost‐injected 3 weeks later with a second dose of these antigens or PBS and observed for another 3 weeks before the MPXV challenge. Following immunization, we found no local inflammatory response and no apparent differences in behavior or body weight among the five groups. As shown in Figure 6A, serum samples were collected 1 day before the second immunization. Remarkably, high levels of neutralizing antibodies were detected in the chemically cross‐linked antigen‐immunized groups, whereas neutralizing antibodies were undetectable in the A29L+A35R‐immunized dormice without chemical cross‐linking or a combination of aluminum adjuvants (Figure 6B). Next, we challenged the immunized dormice with 10^5.5^ PFU MPXV via intranasal inoculation to evaluate the ability of these vaccines to protect against virus replication. The lung tissues of dormice were isolated at 1 week and 2 weeks post‐infection. qRT‒PCR and virus tittering revealed that the viral loads were significantly reduced in all four immunized groups and that both chemical cross‐linking and aluminum enhanced the antiviral efficacy of the antigens (Figure 6C–F). These results demonstrate the enhanced induction of neutralizing antibodies and protection against MPXV replication by chemically cross‐linked antigens, which could be further reinforced by adding an aluminum adjuvant.

### 2.6 Chemically cross‐linked antigens enhanced protection from MPXV infection in vivo

**FIGURE 7:**
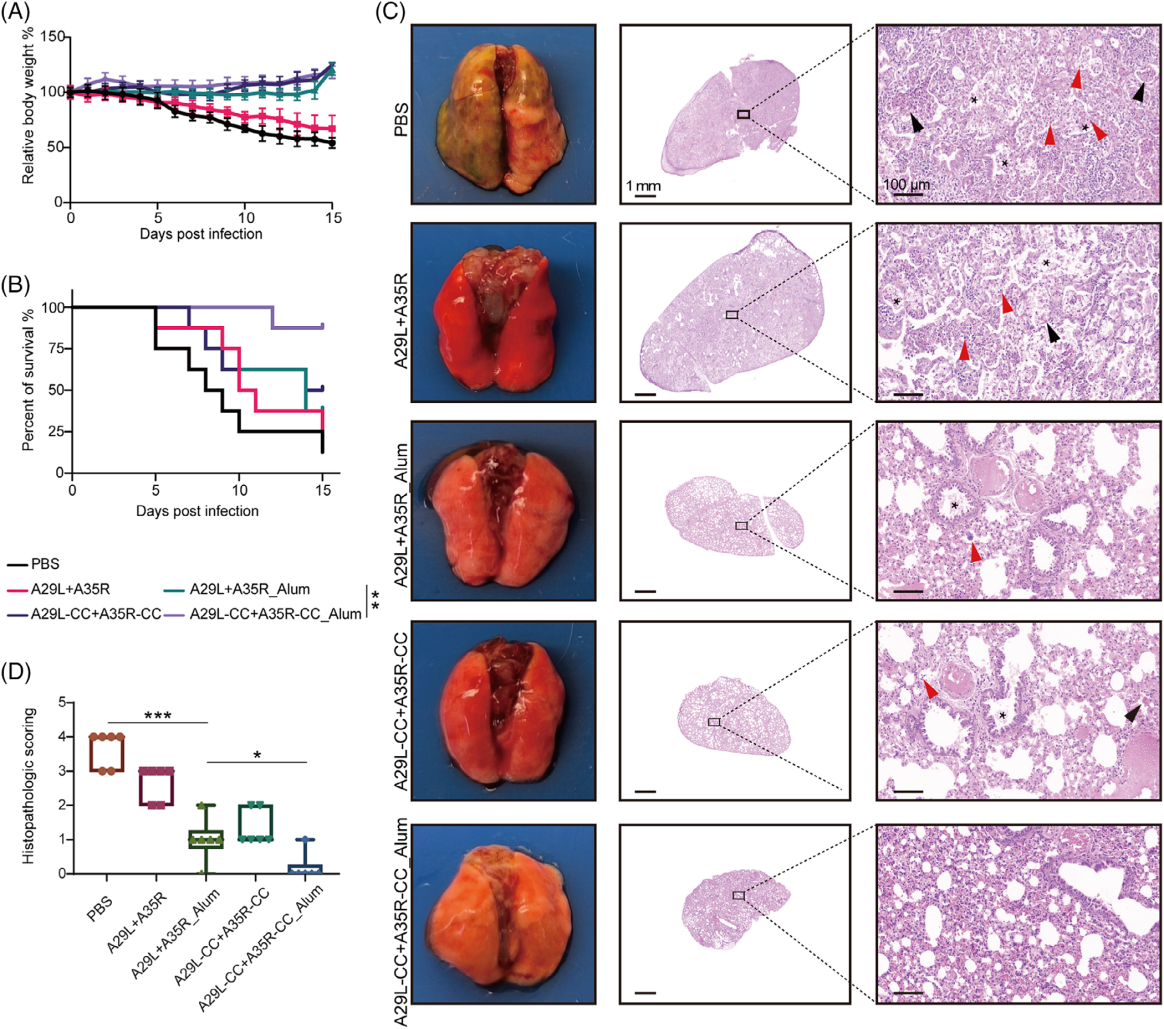
The chemically cross‐linked antigens provided enhanced protection from monkeypox virus infection in vivo. Groups of dormice were immunized with vaccines or placebo and boosted with an equal dose 3 weeks later. After another 3 weeks, the immunized dormice was inoculated with 10^5.5^ monkeypox virus (MPXV) via intranasal injection. The dormice were observed for 15 days for body weight and survival rate analysis and then sacrificed at 14 dpi for pathological analysis of lung damage. (A) Changes in the body weight of MPXV‐infected dormice at 15 dpi. *n* = 8. (B) The percentage of surviving MPXV‐infected dormice at 15 dpi. *n* = 8. (C) Representative histopathological analysis of lung damage in different groups of dormice infected with MPXV at 14 dpi. The black arrows indicate red blood cells that leak into the alveoli; the red arrows indicate inflammatory cell infiltration; the black asterisks indicate cell necrosis and exudate. Scale bar = 1 mm or 100 µm. (D) Histological score based on hematoxylin and eosin (H&E) staining indicating pathological lesions in lung tissues. *n* = 6. The data are expressed as the mean  ±  SEMs. *p < 0.05, **p < 0.01, ***p < 0.001.

To further elucidate the protective effects of chemically cross‐linked antigens on the mortality of dormice subjected to MPXV infection, we monitored the survival rate and body weight changes for 15 days. Notably, MPXV infection induced continuous weight loss in up to 45.79% of dormice, and A29L + A35R immunization alleviated this weight loss to 32.73%. Both aluminum and chemical cross‐linking substantially improved the effectiveness of the A29L + A35R antigens and increased the weight gain of the infected mice (Figure 7A). Moreover, high lethality was observed in the MPXV‐infected dormice, and seven of the nine dormice died from 5 to 15 dpi. As expected, A29L+A35R_Alum and A29L‐CC+A35R‐CC significantly increased the survival rate from 12.5% to 37.5% or 50%, whereas A29L+A35R immunization did not substantially improve the survival rate compared with that of the PBS‐treated group. Notably, A29L‐CC+A35R‐CC_Alum immunization offered potent protection against MPXV damage and substantially elevated the survival rate to 87.5%, suggesting that a synergistic effect was induced by the combination of aluminum and chemical cross‐linking (Figure 7B).

Next, the lung lobes were collected at 14 dpi for systematic pathological analysis. As displayed in Figure 7C, gross pathology revealed severe dark red lesions in large parts of the lung lobes and hemorrhaging in the inferior lobe of the right lung in MPXV‐infected dormice subjected to placebo immunization. Additionally, hematoxylin and eosin (H&E) staining revealed necrotic cell debris and exudate in the bronchial lumens and alveoli (marked by black asterisks), inflammatory cell infiltration in the bronchiolar walls and alveolar interstitium (marked by red arrows), and red blood cell exudation in some alveoli (marked by black arrows) in the model group. In contrast, the A29L+A35R_Alum‐ or A29L‐CC+A35R‐CC‐immunized dormice showed pathological recovery from severe pneumonia and lung injury. Furthermore, combining aluminum and chemically cross‐linked antigens strongly protects against MPXV‐induced pathological damage in the lung lobe. Consistently, the histological scores were markedly greater in the PBS group than in the other groups, and the lungs from the A29L‐CC+A35R‐CC_Alum‐immunized mice had lower histological scores than those from the A29L+A35R_Alum‐ or A29L‐CC+A35R‐CC‐immunized mice (Figure 7D).

## 3 DISCUSSION

Vaccines have been proven to be powerful weapons against infectious pathogens. While combating MPXV, researchers have verified the protective potential of live attenuated vaccinia virus vaccines in mitigating MPXV infection.^20^, ^21^, ^22^ However, given the common coexistence of MPXV infection with HIV infection in the affected population, administering live‐virus vaccines as a preventive measure poses significant risks for immunocompromised individuals.^23^, ^24^, ^25^, ^26^ Therefore, there is an urgent need to develop safer and more effective vaccine strategies to combat MPXV infection. However, unlike attenuated or inactivated vaccines, protein‐based subunit vaccines do not contain viable pathogens, rendering them safer. However, the low immunogenicity of a single antigen limits its effectiveness. In this study, we aimed to develop protein‐based multiantigen vaccines for MPXV.

MPXV has two distinct forms of infectious virus particles, intracellular mature virions (IMVs) and extracellular enveloped virions (EEVs). IMVs and EEVs express different membrane proteins, typically A29L, M1R, E8L, and H3L for IMVs, and B6R and A35R for EEVs, forming unique antigenic features. Therefore, the combination of antigens from IMVs and EEVs is critical for designing vaccines against MPXV. Multiple studies have reported that A35R (A33 VACV) and other antigens form a two‐antigen combination to achieve strong immune effects.^15^ In addition, literature has reported that monoclonal antibodies against A35 (A33 VACV) also have a strong neutralizing effect on VACV.^27^ Similarly, literature reports that monoclonal antibodies against A29L also exhibit good neutralizing effects on MPXV.^28^ Thus, we chose the IMV antigen A29L and the EEV antigen A35R to develop dual‐antigen vaccines for MPXV. However, in our initial trial, the two antigen proteins showed limited uptake by APCs, especially A29L (Figure 4), suggesting that some engineering strategies should be taken to increase the uptake and presentation of the two antigen proteins by APCs. We, therefore, adopted a chemical editing strategy, termed chemical cross‐linking, to engineer protein antigens, aiming to increase antigen uptake and presentation and improve vaccination responses (Figure 1). In this strategy, a bifunctional chemical cross‐linker was utilized to chemically cross‐link the lysine residues with the introduction of disulfide bond linkers, which would be reduced intracellularly by chemicals such as glutathione (GSH), followed by subsequent auto‐elimination reactions to release native antigen proteins in APCs (Figure 2). Chemical cross‐linking led to the formation of protein oligomers (Figures 2 and 3), which resulted in enhanced uptake by APCs (Figures 2 and 4), and the subsequent intracellular release of native proteins guaranteed enhanced antigen presentation (Figure 2). To develop A29L‐ and A35R‐based dual‐antigen vaccines, we explored the chemical cross‐linking strategy by cross‐linking the two proteins individually or jointly. The results showed that individual chemical cross‐linking and separate administration led to the most profound increase in the uptake of the two antigens by APCs (Figure 4), although the exact mechanism underlying this cross‐linking and administration options still needs further investigation. These cross‐linking and administration strategies thus led to increased accumulation of both antigens in the lymph nodes in vivo and increased titers of specific antibodies for both antigens (Figure 5). As an aluminum‐adjuvanted dual‐antigen vaccine, the chemical cross‐linking strategy led to the induction of the highest titer of live virus‐neutralizing antibodies (Figure 6B), further confirming the functions of the chemical cross‐linking strategy. Moreover, we envision that the elevated titer of neutralizing antibodies may target distinct epitopes that may contribute to the neutralization of future variants of interest, which needs to be investigated further.

Encouraged by the above results, we performed MPXV challenge tests after a prime and boost immunization process (Figure 6A). There are various methods for selecting the appropriate dose of vaccine, such as using ED_50_ (the maximum vaccine dose that provides seroconversion in 50% of the animals in a given group) to screen for effective doses.^29^ The antigen immunization dose used in our study was 20 µg, which is consistent with the commonly used 10 and 20 µg in the literature.^30^, ^31^ Moreover, the combination immunization of Mpox A35R and M1R at a dose of 30 µg drove potent immune response (1). Therefore, we have chosen an immunization dose of 20 µg. Under the 20 µg dosage, the aluminum‐adjuvanted chemically cross‐linked dual‐antigen vaccine (A29L‐CC+A35R‐CC_Alum) significantly enhanced immune responses, as evidenced by a reduced virus load (Figure 6C–F), better‐maintained mouse body weights (Figure 7A), and mouse survival (Figure 7B). Notably, gross pathological and H&E staining data from MPXV‐infected dormice revealed severe dark red lesions in most of the lung lobes, hemorrhage in the right lower lung lobes, necrotic cell debris and exudation in bronchial lumens and alveoli, inflammatory cell infiltration in bronchiolar walls and the alveolar interstitium, and red blood cell exudation in some alveoli. In contrast, A29L‐CC+A35R‐CC_Alum‐immunized mice showed pathological recovery following severe pneumonia and lung injury (Figure 7C,D). These findings underscore the robust protective effects of chemically cross‐linked antigen vaccines against pathological damage to the lung lobes.

This work explored the potential of using a reversible chemical cross‐linking approach to develop a dual‐antigen protein vaccine for MPXV. The chemical cross‐linking approach reversibly engineers protein antigens to facilitate their accumulation in lymph nodes and their uptake and presentation by APCs. The aluminum‐adjuvanted chemically cross‐linked dual‐antigen vaccine A29L‐CC+A35R‐CC_Alum thus showed enhanced immune responses in vivo, with the production of higher titers of live virus‐neutralizing antibodies, which in turn led to a reduced virus load in vivo, increased survival and mitigated tissue damage. Although the proof‐of‐concept work in Mpox is limited by restricted antigen presentation (i.e., only two antigens, A29L and A35R, were presented), this work still provides insights for the development of enhanced multiantigen subunit vaccines with the assistance of chemical engineering strategies.

In conclusion, our work presented a reversible chemical cross‐linking strategy to improve the uptake and presentation of protein antigens by APCs. This strategy was applied to a dual‐antigen vaccine against Mpox, and the results showed that the chemical cross‐linking approach led to improved immune responses and increased protection against Mpox in vivo. As a universal strategy, the method provided here may find more applications in the fight against future emerging pathogens.

## 4 MATERIALS AND METHODS

### 4.1 Reagents

Chemical cross‐linker (Xi'an Ruixi Bio), OVA (Thermo Fisher, 77120), Cy3‐NHS (Xi'an Ruixi Bio), Cy5‐NHS (Xi'an Ruixi Bio), Cy7‐NHS (Xi'an Ruixi Bio), PD‐10 column (Cytiva, 17085101), PE anti‐mouse H‐2K^b^ bound to SIINFEKL Antibody (Biolegend, 141603), TMB (Solarbio, PR1200), HRP‐conjugated goat anti‐mouse IgG (ZSGB Bio), and Alhydrogel adjuvant (InvivoGen, vac‐alu‐50).

### 4.2 Viruses

The MPXV virus (Genebank: PP778666.1) used in this study was isolated from a patient in Guangzhou, China. The virus was propagated in Vero E6 cells and cultured in Dulbecco's minimal essential medium (DMEM) (Sigma‐Aldrich) containing 10% fetal bovine serum (Invitrogen), 50 U mL^−1^ of penicillin, and 50 µg mL^−1^ of streptomycin. All experiments with infectious MPXV virus were conducted in a biosafety level 3 laboratory.

### 4.3 Construction, expression, and purification of A29L and A35R

The genome information of MPXV “Singapore 2019” strain was used as a reference, and the DNA sequences coding for A29L (QJQ40281.1) and A35R (QJQ40286.1) proteins were obtained from NCBI protein database (https://www.ncbi.nlm.nih.gov/protein/). A DNA fragment coding full‐length A29L or A35R proteins, with transmembrane domain (V32‐V57 aa) deleted, was synthesized by General Biol. Inc., and inserted into the pET30a vector. 6xHis Tag was fused in the C‐terminal of recombinant protein. Recombinant protein was expressed in *E. coli* BL21(DE3) cells, and the expression of each recombinant protein was induced using 1 mM IPTG (Sangon Biotech). Cells were harvested at 6‐h post‐induction of IPTG and lysed by homogenization (ATS Engineering Inc.). The fraction of supernatant or pellet from the cell lysate was separated by centrifugation at 12,000 rpm for 30 min at 4°C. A29L was present in a soluble form in the supernatant and could be purified by Ni affinity chromatography directly. But A35R was insoluble and existed in the form of inclusion body. The A35R inclusion body was resuspended and denatured with 20 mM Tris‐HCl buffer (containing 8 M urea) after washing three times with IB washing buffer (50 mM Tris–HCl, 50 mM NaCl, 5 mM EDTA, 10% (w/v) glycerine, 0.3% sodium deoxycholate, 5% Triton X‐100, pH 8.0). The solution of denatured A35R was added dropwise into a 20‐fold volume of the refolding buffer (20 mM Tris‐HCl, 20 mM NaCl, 0.2 M arginine, 5 mM EDTA, 1 mM oxidized glutathione, 10% (w/v) glycerine, pH 10) with mild stirring. The refolded A35R was dialyzed against the loading buffer (20 mM Tris‐HCl, 150 mM NaCl, pH 8.5). The His‐Trap HP column (5 mL, GE Healthcare) and an Akta Pure chromatography system (Cytiva) was used to purify the recombinant proteins. The purity of A29L or A35R was analyzed by SDS–PAGE.

### 4.4 Chemical cross‐linking of OVA, A29L and A35R

For OVA cross‐linking, 5 µM of OVA was mixed with 500 µM chemical cross‐linker molecule. For A29L‐CC and A35R‐CC, 20 µM of monomeric proteins was mixed with either 1 or 2 mM chemical cross‐linker molecule. For A29L‐A35R‐CC, 10 µM of each monomeric protein was mixed with either 1 or 2 mM chemical cross‐linker molecule. The reactions were incubated at 30°C for 1 h with continuous shaking. The reaction mixture was purified with PD‐10 desalting column to remove excess amount of residue chemical cross‐linker molecule. For in vitro uptake experiments and in vivo lymph node accumulation experiments, proteins were pre‐labeled with low equivalent fluorescence dye (Cy3, Cy5, or Cy7) with NHS functionalized dyes. The cross‐linked proteins were stored at −80°C until use.

### 4.5 Dynamic light scattering

OVA and OVA‐CC were diluted in PBS at a concentration of about 0.2 mg/mL. DLS tests were performed using Zetasizer Nano ZS‐90.

### 4.6 SDS–PAGE

Fluorescence dye labeled A29L, A35R, A29L‐CC, A35R‐CC, and A29L‐A35R‐CC were analyzed by SDS–PAGE under reducing (reduced by DTT containing SDS–PAGE loading buffer) or non‐reducing conditions (SDS–PAGE loading buffer without DTT). The gels were imaged in Cy3 and Cy5 channels followed by Commassie brilliant blue staining. Cross‐linking bands were visualized under nonreducing condition and broke down under reducing condition.

### 4.7 Flow cytometry analysis

To visualize the antigen protein uptake by APCs, fluorescence dye labeled monomeric or cross‐linked antigens (equivalent to 100 nM dyes) were incubated with dendritic cells (DC2.4) and macrophages (RAW264.7) for 4 h. Flow cytometry analysis was conducted with Bio‐Rad S3e (Bio‐Rad) under Cy3 or Cy5 channel. To visualize the antigen‐presenting of OVA, DC2.4 cells were first incubated with OVA or OVA‐CC (200 nM) for 8 h. Cells were then stained with PE anti‐mouse H‐2K^b^ bound to SIINFEKL Antibody and analyzed using Bio‐Rad S3e (Bio‐Rad) under PE channel.

### 4.8 Confocal microscopy

For confocal microscopy imaging, dendritic cells (DC2.4) and macrophages (RAW264.7) were treated with cross‐linked antigen as described for flow cytometry analysis. After 4‐h incubation, cells were washed three times with PBS and then stained with Hoechst 33342. The images were taken with Zeiss LSM 900 in the channels of Hoechst and Cy3 or Cy5.

### 4.9 Validation of antigen proteins accumulation in inguinal lymph nodes

Cy5‐labeled A29L or A29L‐CC and Cy7‐labeled A35R or A35R‐CC (equivalent to 1 nmol dyes) were administrated to Balb/c mouse via intramuscular injection. Twenty‐four hours later, mouse was sacrificed and inguinal lymph nodes were collected. Ex vivo imaging of the inguinal lymph nodes performed with AniView 600 instrument (Guangzhou Biolight Biotechnology). A29L and A29L‐CC accumulation in inguinal lymph nodes was analyzed under Cy5 channel. A35R and A35R‐CC accumulation in inguinal lymph nodes was analyzed under Cy7 channel.

### 4.10 In vivo immunization for specific antibody tests

Dormouse was prime immunized with 20 µg A29L and A35R or 20 µg A29L‐CC and A35R‐CC intramuscularly at day 0 and boosted with same amount of antigens at day 21. Mouse serum was collected and stored at −80°C at day 33 for before validation of specific antibodies with ELISA.

### 4.11 Measurement of specific antibodies using ELISA

A29L or A35R (1 µg/mL) was coated on EIA/RIA plates (Corning) at 4°C overnight. The plates were washed twice with PBST (0.5% Tween‐20 in PBS) and then blocked with 2% BSA in PBS for 2 h at room temperature. The plates were further washed twice with PBST. The collected serum was serial diluted with PBST‐BSA (PBST with 0.5% BSA). Diluted serum was incubated with A29L‐ or A35R‐coated EIA/RIA plates at room temperature for 1 h before washed with PBST for three times. HRP‐conjugated goat anti‐mouse IgG was diluted with PBST (1:5000), added to the plates, and incubated for 1 h at room temperature. The plates were washed with PBST for three times. TMB solution (100 µL/well) was added and the plates were kept at dark for about 30 min and then stop with Stop solution (50 µL/well). Absorbance at 450 nm was measured immediately to determine the levels of specific antibodies.

### 4.12 In vivo immunization for virus challenge

Dormouse was immunized with 20 µg A29L and A35R or 20 µg A29L‐CC and A35R‐CC with or without 40 µg of aluminum hydroxide adjuvant. Mouse was prime and boost at week −6 and week −3. Serum was first collected 1 day before boost to determine early neutralizing antibodies. Mouse was challenged with MPXV at week 0 and lung tissues were collected 1 and 2 weeks after virus challenge. Blood samples were subjected to virus load validation.

### 4.13 Neutralizing antibody tests

An authentic MPXV neutralization assay was performed using the cytopathic effect assay. Serial dilutions of the serum samples were prepared in 96‐well plates by a factor of two and then incubated with an equal volume of 100 PFU of MPXV virus at 37°C for 1 h. Thereafter, Vero E6 cells were seeded at a density of 5 × 10^3^ cells/well in a plate and cultured for 4 days at 37°C. The resulting cytopathic effects caused by the virus were recorded and the percentage of neutralization was calculated at a specific antibody concentration based on the obtained results. The average ± SD (triplicates) or averages (duplicates) were plotted using nonlinear regression (log [inhibitor] vs. normalized response, variable slope).

### 4.14 MPXV challenge

Dormice were intranasally inoculated with 10^5.5^ PFU MPXV in a volume of 100 µL. Body weight and survival rate were recorded for a total of 15 days. The dormice were sacrificed on 7 and 14 dpi and lung lobes were isolated for quantitative real‐time PCR (qRT‐PCR) analysis, plaque reduction neutralization test, and histopathological examination.

### 4.15 qRT‐PCR analysis

Viral DNA in the lung tissue homogenate was isolated and processed with E.Z.N.A. Viral DNA Kit (OMEGA, D3892, USA) following the manufacturer's instructions. Viral copy numbers were detected by absolute qRT‐PCR using absolute quantitative qRT‐PCR methodology using the Eastep qPCR Master Mix (Promega, SL2062) and an ABI 7500 real‐time PCR system (Applied Biosystems). The protocol for the qRT‐PCR was as follows: 95°C for 2 min, followed by 40 cycles at 95°C for 15 s and 60°C for 1 min. The specific primers used to detect the MPXV F3L genes were as follows:

F3L gene

Forward: 5′‐CATCTATTATAGCATCAGCATCAGA‐3′;

Reverse: 5′‐GATACTCCTCCTCGTTGGTCTAC‐3′;

### 4.16 Plaque reduction neutralization test

The neutralizing antibody titers were determined by plaque reduction neutralization test (PRNT50) on 3 weeks after vaccination of mice. Vero E6 cells were seeded in 12‐well plates, and twofold serial dilutions of serum samples were mixed with an equal volume of MPXV containing −100 PFU of virus per milliliter; the virus/serum mixtures were added to wells of the 12‐well plates containing Vero E6 cell culture monolayers. The plates were then incubated at 37°C for 90 min. Cells were overlaid with 0.5% agarose in DMEM (Gibco) with 2.5% inactivated FBS. After incubated at 37°C in 5% CO_2_ for 72 h, cells were fixed by 4% formaldehyde and stained by 0.2% crystal violet, and then the Plaque numbers were recorded. The calculation of PRNT50 has described previously.^32^, ^33^, ^34^

### 4.17 Histopathological examination

Lung tissues were fixed in 4% paraformaldehyde solutions for 7 days, paraffin‐embedded, sectioned, and stained with H&E according to standard protocols. Images were captured using light microscopy. The pathology scoring of lung tissue follows the established method. The main pathological changes observed in the lungs include degeneration, necrosis, and shedding of bronchial epithelial cells, inflammatory cell infiltration in the bronchial peribronchial and alveolar interstitium, and exudate in the bronchial and alveolar cavities. To more accurately assess the severity of the lesions, we scored the lesions in each group of lung tissues based on the extent of involvement as follows: a score of 0 indicates no lesions in the lung tissue, a score of 1 indicates lesions involve less than 10% of the affected area, a score of 2 indicates lesions involve 10%–50% of the affected area, a score of 3 indicates lesions involve 50%–90% of the affected area, and a score of 4 indicates lesions involve over 90% of the affected area.

### 4.18 Statistical analysis

Statistical significance between groups was determined using GraphPad Prism, Version 8.0. Data were presented as mean ± SD or mean ± SEM in all experiments and analyzed using a *t‐*test or analysis of variance followed by a two‐tailed *t*‐test, and a *p *< 0.05 was considered to be statistically significant.

## AUTHOR CONTRIBUTIONS

**Long Chen; Chao Shang; Zihao Wang; Yunsheng Yuan; Shiyong Fan; Wu Zhong; Jian Lin; and Xiao Li**: Conceptualization. **Long Chen; Chao Shang; Zihao Wang; Mengzhu Zheng; Cuiling Zhang; Dapeng Li; Zhanqun Yang; Yuchao Dong; and Yuru Xu**: Investigation. **Long Chen; Chao Shang; Zihao Wang; Mengzhu Zheng; and Yunsheng Yuan**: Visualization. **Xiao Li**: Funding acquisition. **Long Chen; Chao Shang; and Zihao Wang**: Project administration. **Yunsheng Yuan; Shiyong Fan; Wu Zhong; Jian Lin; and Xiao Li**: Supervision. **Long Chen; Chao Shang; Zihao Wang; and Mengzhu Zheng**: Writing—original draft. **Yunsheng Yuan; Shiyong Fan; Wu Zhong; Jian Lin; and Xiao Li**: Writing—review and editing. All authors have read and approved the final manuscript.

## CONFLICT OF INTEREST STATEMENT

The authors declare no conflicts of interest.

## ETHICS STATEMENT

In this study, the virus and animal studies were approved by the ethics committee of Changchun Veterinary Research Institute (Approval number: IACUC of AMMS‐11‐2024‐028).

## Supporting information



Supporting information

## Data Availability

The data are available from the corresponding author on reasonable request.
