# Supplementary material for: Chemical cross‐linking facilitates antigen uptake and presentation and provides improved protection from Mpox with a dual‐antigen subunit vaccine
Source: MedComm (2020). 2025 Jan 8;6(1):e70045. doi: 10.1002/mco2.70045 (PMC11707423; doi:10.1002/mco2.70045)
Supplement: Supplementary file 1 — Supporting information [file MCO2-6-e70045-s001.docx]

**Supplementary Information**

**Chemical crosslinking facilitates antigen uptake and presentation and provides improved protection from Mpox with a dual-antigen subunit vaccine**

**Long Chen^1,#^, Chao Shang^2,#^, Zihao Wang^3,#^, Mengzhu Zheng^4,#^, Cuiling Zhang^2^, Dapeng Li^2^, Zhanqun Yang^1^, Yuchao Dong^3^, Yuru Xu^3^, Yunsheng Yuan^5,*^, Shiyong Fan^3,*^, Wu Zhong^3,*^, Jian Lin^1,4,*^, Xiao Li^2,*^**

^1^Department of Pharmacy, Peking University Third Hospital Cancer Center, Peking University Third Hospital, Beijing 100191, China

^2^Changchun Veterinary Research Institute, Chinese Academy of Agricultural Sciences, Changchun 130122, China

^3^National Engineering Research Center for the Emergency Drug, Beijing Institute of Pharmacology and Toxicology, Beijing 100850, China

^4^Key Laboratory of Tropical Biological Resources of Ministry of Education, Song Li's Academician Workstation of Hainan University, School of Pharmaceutical Sciences, Hainan University, Haikou 572000, China

^5^Engineering Research Center of Cell & Therapeutic Antibody, Ministry of Education, Shanghai Jiao Tong University School of Pharmacy, Shanghai 200240, China

^*^**Correspondence**

Yunsheng Yuan, Engineering Research Center of Cell & Therapeutic Antibody, Ministry of Education, Shanghai Jiao Tong University School of Pharmacy, Shanghai 200240, China.

Email: yunsheng@sjtu.edu.cn (Yunsheng Yuan)

Shiyong Fan, National Engineering Research Center for the Emergency Drug, Beijing Institute of Pharmacology and Toxicology, Beijing 100850, China.

Email: fansy@bmi.ac.cn (Shiyong Fan),

Wu Zhong, National Engineering Research Center for the Emergency Drug, Beijing Institute of Pharmacology and Toxicology, Beijing 100850, China.

Email: zhongwu@bmi.ac.cn (Wu Zhong),

Jian Lin, Department of Pharmacy, Peking University Third Hospital Cancer Center, Peking University Third Hospital, Beijing 100191, China.

Email: linjian@pku.edu.cn (Jian Lin),

Xiao Li, Changchun Veterinary Research Institute, Chinese Academy of Agricultural Sciences, Changchun 130122, China.

Email: skylee6226@163.com (Xiao Li)

**^#^**These authors are co-first author


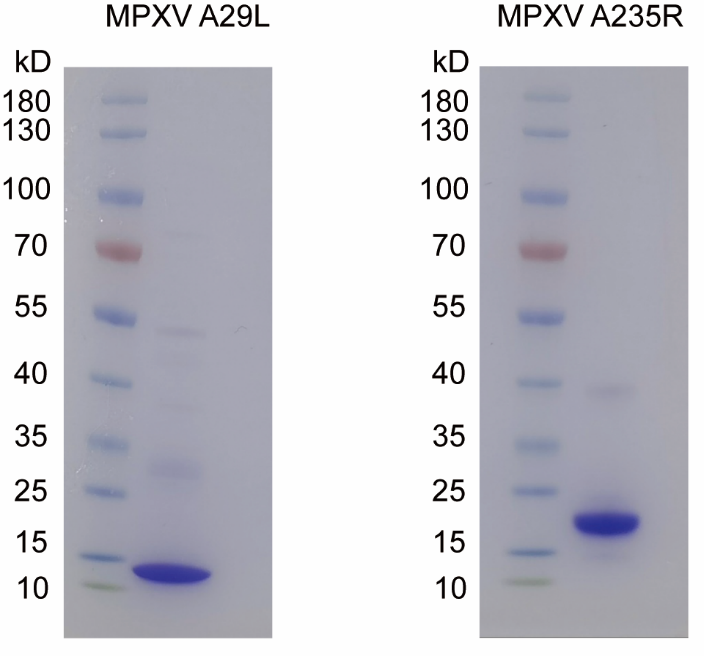


**Figure S1.** SDS-PAGE analysis of purified recombinant monkeypox virus A29L and A35R proteins.


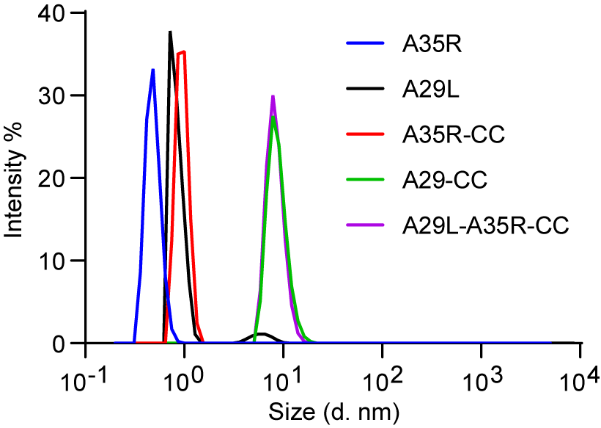


**Figure S2.** Dynamic light scattering analysis of A29L, A35R and chemical crosslinked proteins. Diameter: A35R 0.49 nm, A35R-CC 0.96 nm, A29L-CC 8.8 nm, A29L-A35R-CC 8.4 nm. PDI (Polydispersity Index): A29L 0.428, A35R 0.271, A35R-CC 0.55, A29L-CC 0.608, A29L-A35R-CC 0.495.


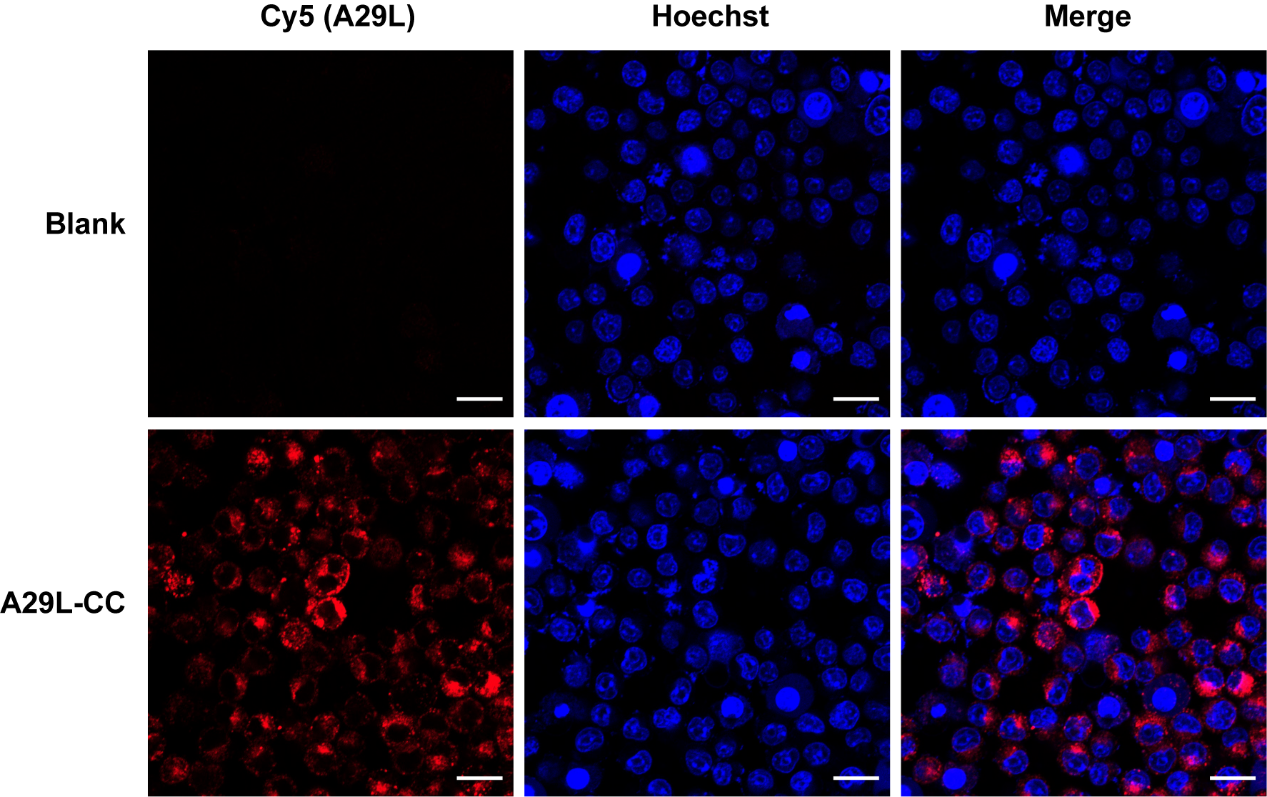


**Figure S3.** Confocal microscope imaging showing the internalization of A29L-CC by dendritic cells. Scale bars: 20 μm. Red: A29L-CC labeled with Cy5 dye. Blue: nucleus stained with Hoechst 33342 dye.


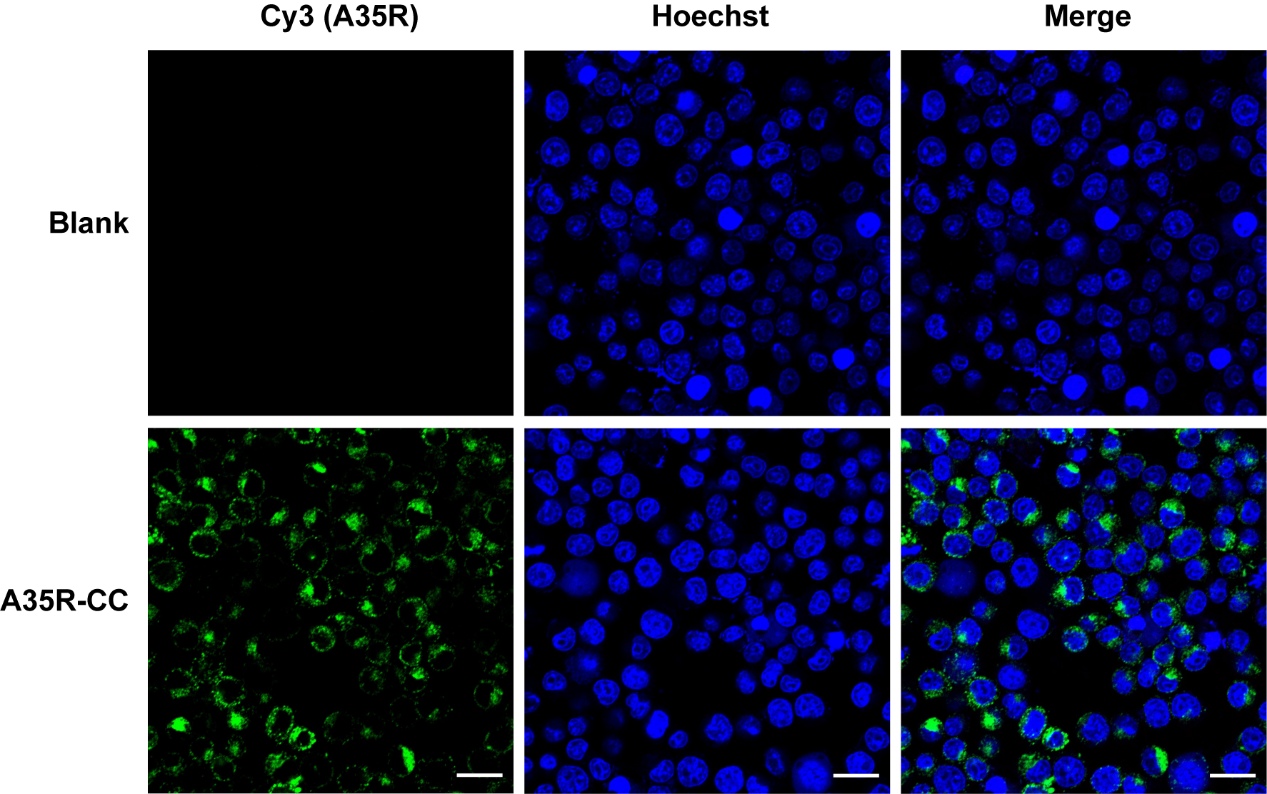


**Figure S4.** Confocal microscope imaging showing the internalization of A35R-CC by dendritic cells. Scale bars: 20 μm. Green: A35R-CC labeled with Cy3 dye. Blue: nucleus stained with Hoechst 33342 dye.


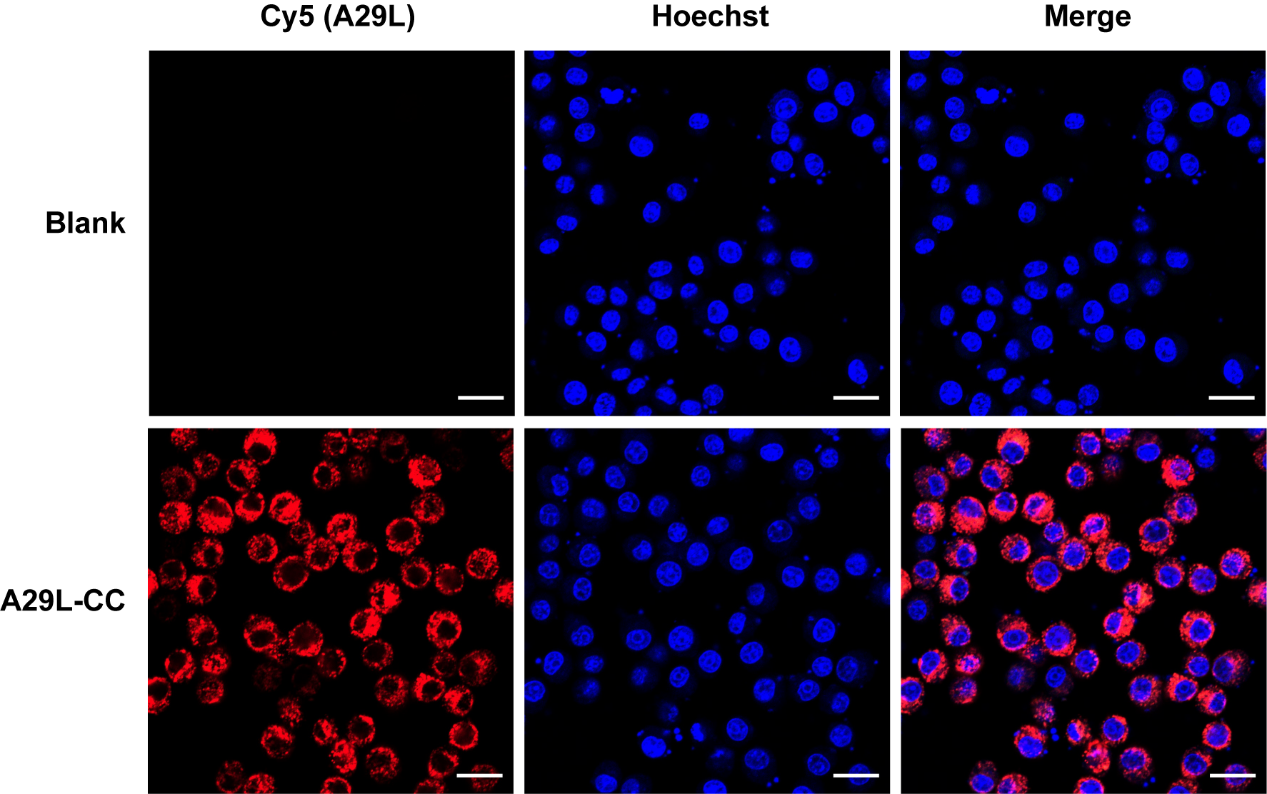


**Figure S5.** Confocal microscope imaging showing the internalization of A29L-CC by macrophages. Scale bars: 20 μm. Red: A29L-CC labeled with Cy5 dye. Blue: nucleus stained with Hoechst 33342 dye.


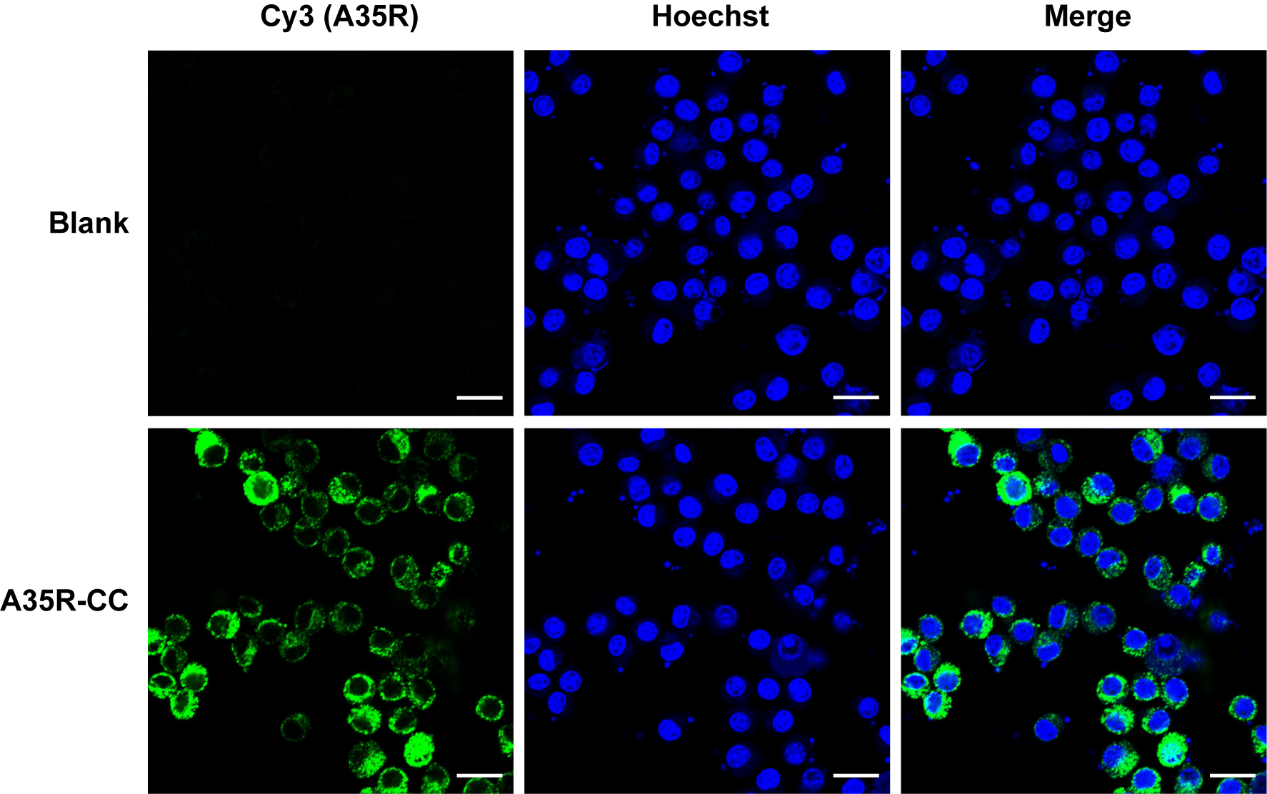


**Figure S6.** Confocal microscope imaging showing the internalization of A35R-CC by macrophages. Scale bars: 20 μm. Green: A35R-CC labeled with Cy3 dye. Blue: nucleus stained with Hoechst 33342 dye.
